# Health Risk Evaluation of Trace Elements in Geophagic Kaolinitic Clays within Eastern Dahomey and Niger Delta Basins, Nigeria

**DOI:** 10.3390/ijerph17134813

**Published:** 2020-07-04

**Authors:** Olaonipekun Oyebanjo, Georges-Ivo Ekosse, John Odiyo

**Affiliations:** 1 Natural History Museum, Obafemi Awolowo University, Ile-Ife 220282, Osun State, Nigeria; 2 Directorate of Research and Innovation, University of Venda, Thohoyandou 0950, Limpopo Province, South Africa; ekosseg@gmail.com; 3 School of Environmental Sciences, University of Venda, Thohoyandou 0950, Limpopo Province, South Africa; John.odiyo@univen.ac.za

**Keywords:** geophagia, health risk, kaolin, dietary intake, pollution

## Abstract

The deliberate consumption of earthly materials is a universally recognised habit with health benefits and risks to those that practice it. Thirteen (13) samples comprising of six (6) Cretaceous and seven (7) Paleogene/Neogene geophagic kaolinitic materials, respectively, were collected and analysed for trace element concentrations (V, Cr, Co, Ni, Zn, Pb, and Fe), and possible risk on consumers’ health. The trace element compositions were obtained using laser ablation inductively coupled plasma mass spectrometry (LA-ICPMS) and X-ray fluorescence spectrometry (XRF) analytical methods. Based on their average concentrations, Fe > V > Cr > Ni > Zn > Pb > Cu > Co and Fe > V > Cr > Zn > Cu > Pb > Ni > Co for the Cretaceous and Paleogene/Neogene geophagic clays, respectively. Iron concentrations were significantly higher in Paleogene/Neogene geophagic clays than in Cretaceous geophagic clays. The nutritional value of Cu and Zn were lower whereas, Cr and Fe were higher than the recommended dietary intake. The index of geoaccumulation (0 < I_geo_ ≤ 1) showed that the geophagic materials were uncontaminated to moderately contaminated by the trace elements. The overall hazard indices (HI) for non-carcinogenic effects showed that the geophagic clays pose threat to children (HI > 1) and no threat to adults (HI < 1) health. However, the carcinogenic risk indices (CRI) for Cr, Ni, and Pb were within acceptable cancer risks (10^−6^ < CRI < 10^−4^) for children and adults. Hence, based on the trace element s HI and CRI, this study concluded that the consumption of Cretaceous and Paleogene/Neogene geophagic kaolinitic clays poses no risks to adult health but children might suffer health risk if the geophagic clays are not beneficiated before ingestion.

## 1. Introduction

Geophagy among humans dates to several centuries ago, it is widely practiced in various parts of the world [1,2]. It has been reported in several countries in Africa such as Nigeria, South Africa, Kenya, Ghana, Cameroon, etc. [3,4,5]. The first documentation of geophagic practice in Nigeria dates to the 1960s among TIV pregnant women in Benue State [6,7]. It is prevalent among the Yoruba, Hausa, and Igbo tribes in Nigeria [8]. There are several reasons why people from diverse age groups, gender, status, and background practice geophagia. Some of these reasons could be cultural, as people feel it is a blessing from their ancestors and eating it will allow fertility in women [9]. For religious reasons, people practice geophagia as a form of spiritual ritual [10]. For example, children suck cakes with Christ impressions around the United States–Mexican border towns [11]. Often, medical reasons prevail most, either for supplementary mineral nutrients (such as calcium (Ca), iron (Fe), and magnesium (Mg)) [12] or detoxifying poisonous substances because of the high cationic exchange capacity of the clays [13]. Women in Africa believe that eating earth material could reduce nausea and vomiting during pregnancy [14]. Geophagic practice could be passed from parents to children or unintentionally by ingesting earth materials while playing on the ground or floor by hand to mouth movements [15].

Kaolin minerals dominate the clay minerals in geophagic materials with various concentrations of major and trace elements which have health implications [16]. Quartz occurring with kaolinite in most geophagic materials could cause dental enamel damage, abrasion of the gastrointestinal tract, and rupturing of the colon [17]. The red colour of the geophagic soils commonly consumed is usually due to the presence of Fe [18] which may not be bioavailable [19]. The genesis and properties of the geophagic material determines its influence on human health. In recent times, health risks associated with trace elements present in several geophagic materials have drawn the attention of medical geologists and environmental health scientists. The impact of trace elements toxicity levels on human health depends on the type of element and its concentration in the material [5]. For example, higher concentrations of Fe in the human body are known to cause hepatic failure and arthritis [20,21], whereas renal dysfunction, nausea, and diarrhea have been linked to excessive copper (Cu), cadmium (Cd), and arsenic (As) [21]. Toxicity of lead (Pb) means it is harmful for babies in the womb and after birth because it can result in birth defects and brain damage [8]. In addition, high levels of chromium (Cr) tend to cause kidney failure, dermatitis, and pulmonary cancer [22,23].

Previous studies on the health risks associated with the consumption of geophagic materials have only reported the concentrations of trace elements and its health implications relative to global averages and World Health Organisation (WHO) standards [3,4,24,25]. This approach does not examine potential non-carcinogenic and carcinogenic risks to humans [26]. However, few studies have assessed the non-carcinogenic and carcinogenic health risks associated with trace elements in geophagic samples [5,8,27,28] through direct oral ingestion, inhalation, and dermal absorption based on the guidelines put forward by the United States Environmental Protection Agency [29]. Lar et al. [8] is one of the few studies on geophagic clay materials in Nigeria that assessed the potential health risks associated with trace elements present in them. They reported high risk to human health particularly pregnant women and children due to the presence of As, Cd, Pb, selenium (Se), and antimony (Sb). Okunlola and Owoyemi [4] concluded that manganese (Mn), Cr, nickel (Ni), and cobalt (Co) concentrations were higher when compared to global occurrences in some geophagic clays from Southern Nigeria. More studies are required to assess the statuses of several other geophagic materials and report potential health risk associated with human consumption for a healthier populace. Hence, this study aimed at establishing the concentrations of selected trace elements in Cretaceous and Paleogene/Neogene geophagic kaolinitic clays from Eastern Dahomey and Niger Delta Basins, Nigeria as well as estimating the possible associated health risks to humans.

## 2. Materials and Methods

**Figure 1:**
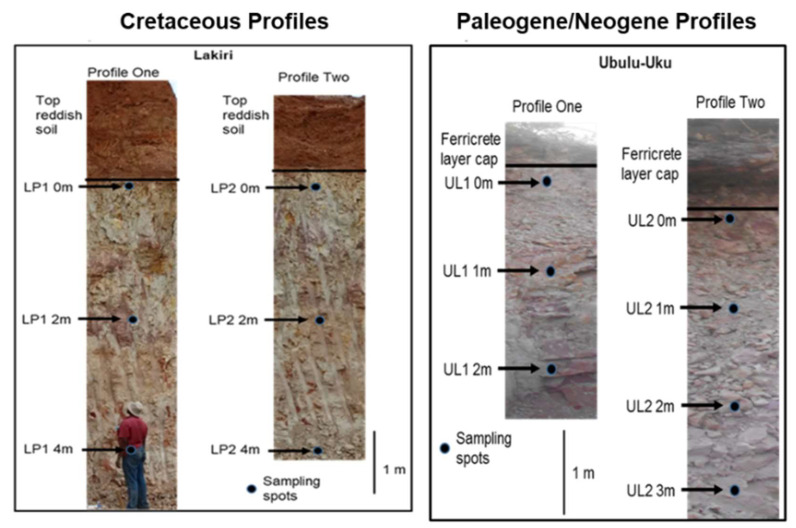
Vertical profiles showing sampling spots and lithologic units of the studied geophagic kaolinitic clay deposits (Modified from [30]).

**Figure 2:**
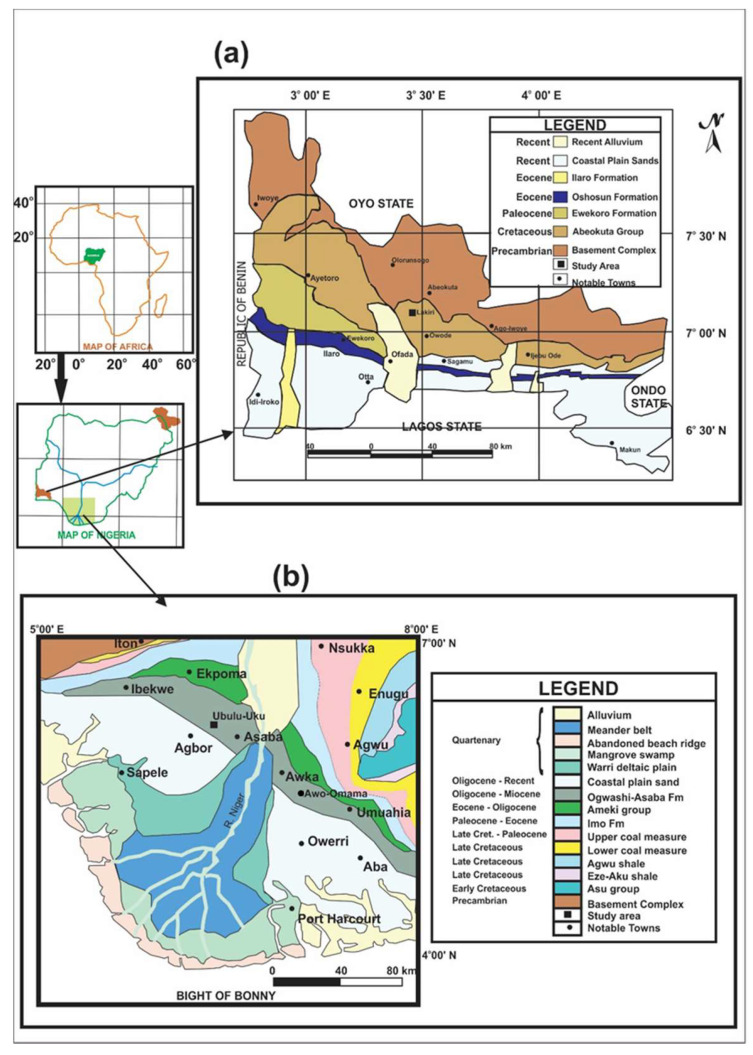
Geologic Maps of (**a**) Eastern Dahomey and (**b**) Niger Delta Basins showing the study areas (Modified after [30,40]).

A total of thirteen (13) geophagic kaolinitic clays comprising of six (6) Cretaceous and seven (7) Paleogene/Neogene were collected from Lakiri and Ubulu-Uku deposits within Eastern Dahomey and Niger Delta Basins, Nigeria, respectively (Figure 1 and Figure 2). The dominance of kaolins in the clays have been established through a separate study [30], hence, the term ‘kaolinitic clays’ is used to define rocks rich in kaolins without particle size connotation [31].

Fresh samples were collected at 2 m and 1 m intervals from Lakiri (LP) and Ubulu-Uku (UL) deposits within Ise (Abeokuta Group) and Ogwashi-Asaba Formations from outcrop faces dug to 60 cm (Figure 1 and Figure 2). Detailed geologic descriptions of the formations have been published earlier by Oyebanjo et al. [32]. The samples were air-dried and gently pulverised to a finer homogenous grain size prior to analyses. No sieving was carried out to have the samples in the state in which they are consumed [3].

The trace elemental concentrations of vanadium (V), Cr, zinc (Zn), Cu, Pb, Ni, and Co in the geophagic samples were determined using an Excimer laser with 193 nm resolution attached to an agilent 7700 series inductively coupled plasma mass spectrometer (Agilent Technologies, Santa Clara, CA, USA) at the Central Analytical Facilities (CAF), Stellenbosch University (SU), South Africa (SA). Certified reference Basaltic Hawaiian Volcanic Observatory (BHVO) glass and powder by United States Geological Survey (USGS) were used as standards [33,34]. The limit of detection (LOD) and limit of quantification (LOQ) for each element in mg kg^−1^ were 0.015 and 0.20, 0.148 and 1.99, 0.011 and 0.23, 0.101 and 1.52, 0.130 and 1.56, 0.004 and 0.06, and 0.008 and 0.11 for V, Cr, Cu, Ni, Zn, Pb, and Co, respectively. The analytical procedures and processing followed those described by Adebayo et al. [35]. Glitter software [36], distributed by Access Macquarie Ltd., Macquarie University, New South Wales, was used for data processing. A PANalytical Axios Wavelength Dispersive X-ray Fluorescence Spectrometer (Malvern Panalytical Ltd, Malvern, United Kingdom) at CAF, SU, SA equipped with a 2.4 kW Rhodium tube was used to determine Fe concentrations (detection limit of 0.5 mg kg^−1^) in fused glass disks. Theoretical alpha and measured line overlap factors to the raw intensities measured with the SuperQ PANalytical software were used to correct for matrix effects in the samples. The NIM-G (Bushveld Granite from the Council for Mineral Technology, South Africa) and BE-N (Basalt from the International Working Group) were used as standards. The analytical procedures and processing followed those described by Fitton [37].

Pollution levels and risk evaluation due to the consumption of the geophagic kaolinitic clays were conducted using the index of geoaccumulation (I_geo_), hazard index (HI), and carcinogenic risk index (CRI), respectively.

The I_geo_ developed by Muller [38] was computed following the formula:

I_geo_ = log_2_ [(C_n_/1.5B_n_)] (1)

where C_n_ is the concentration of the trace element, B_n_ is the background of the element using the upper continental crust (UCC) values [39], and the factor 1.5 was used to minimise the possible variations in B_n_ due to lithogenic influence. The I_geo_ descriptive classes for the contamination level are as follows: uncontaminated (I_geo_ ≤ 0), uncontaminated to moderate (0 < I_geo_ ≤ 1), moderate (1 < I_geo_ ≤ 2), moderate to heavy (2 < I_geo_ ≤ 3), heavy (3 < I_geo_ ≤ 4), heavy to extremely (4 < I_geo_ ≤ 5), and extremely (I_geo >_ 5) [38].

The HI and CRI were used to estimate the non-carcinogenic and carcinogenic risks associated with the individual trace elements through direct oral ingestion, inhalation of particulate elements through mouth and nose, and dermal absorption of the elements on exposed skin [24,41,42]. HI is the sum of hazard quotients (HQ) for each of the pathways (ingestion (HQ_ing_), inhalation (HQ_inh_), and dermal (HQ_derm_)) whereas, CRI is the sum of the chronic daily intake (CDI, mg kg^−1^ d^−1^) for each of the pathways (ingestion (CDI_ing_), inhalation (CDI_inh_), and dermal (CDI_derm_). The CDI, HI, and CRI values for the individual elements through the pathways were computed as follows:

(2) $$CDI_{ing}=\frac{C\times IngR\times EF\times ED\times CF}{BW\times AT}$$

(3) $$CDI_{inh}=\frac{C\times InhR\times EF\times ED}{PEF\times BW\times AT}$$

(4) $$CDI_{derm}=\frac{C\times SA\times AF\times ABS\times EF\times ED\times CF}{BW\times AT}$$

(5) $$HI=\sum_{i=n}^{n}HQ=\sum_{i=1}^{n}\left(\frac{CDI_{ing}^{i}}{RfD_{ing}^{i}}\right)+\sum_{i=1}^{n}\left(\frac{CDI_{inh}^{i}}{RfD_{inh}^{i}}\right)+\sum_{i=1}^{n}\left(\frac{CDI_{derm}^{i}}{RfD_{derm}^{i}}\right)$$

(6) $$CRI=\sum_{i=1}^{n}\left(CRI_{ing}^{i}+CRI_{inh}^{i}+CRI_{derm}^{i}\right)$$

$$=\sum_{i=1}^{n}\left(CDI_{ing}^{i}\times SF_{ing}^{i}\right)+\sum_{i=1}^{n}\left(CDI_{inh}^{i}\times SF_{inh}^{i}\right)+\sum_{i=1}^{n}\left(CDI_{derm}^{i}\times SF_{derm}^{i}\right)$$

Details of the parameters used in Equations (2)–(6) are presented in Table 1. HI values < 1 suggest no significant risk of non-carcinogenic effects, whereas HI > 1 suggest potential risk of non-carcinogenic effects [42,43]. In addition, CRI values > 1 × 10^−4^ is considered unacceptable with potential adverse carcinogenic risk to human health, whereas values between 1 × 10^−6^ and 1 × 10^−4^ are generally acceptable [42,44].

A T-test was used to assess the differences in the trace element concentrations between the Cretaceous and Paleogene/Neogene geophagic kaolinitic clays using IBM Statistical Package for Social Sciences (SPSS) version 25.0.

## 3. Results and Discussion

**Figure 3:**
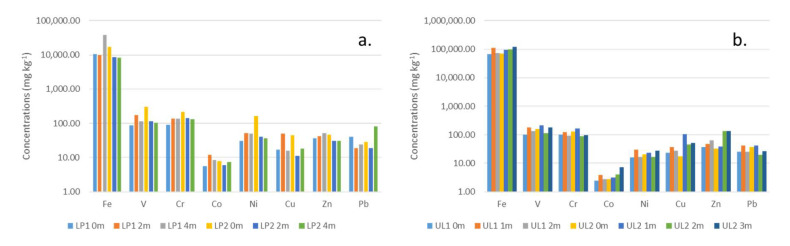
Trace element variations in Cretaceous (**a**) and Paleogene/Neogene (**b**) geophagic kaolinitic clays from Eastern Dahomey and Niger Delta Basins, Nigeria.

The results of trace element concentrations in the geophagic kaolinitic clays are presented in Table 2. In the Cretaceous geophagic kaolinitic clays, the mean Fe, V, Cr, Co, Ni, Cu, Zn, and Pb were 15,560.22, 149.82, 143.54, 7.92, 62.42, 26.17, 39.91, and 35.16 mg kg^−1^, respectively. No unique trace element concentration variations with depth was observed except for LP2 0m having the highest concentrations (except for Pb) (Figure 3a). Mean values for Fe, V, Cr, Co, Ni, Cu, Zn, and Pb were 90,685.70, 155.08, 113.45, 3.77, 21.68, 43.80, 69.85, and 30.96 mg kg^−1^, respectively in the Paleogene/Neogene geophagic kaolinitic clays. Samples from UL2 1m and 3m had higher trace element concentrations relative to others (Figure 3b).

**Figure 4:**
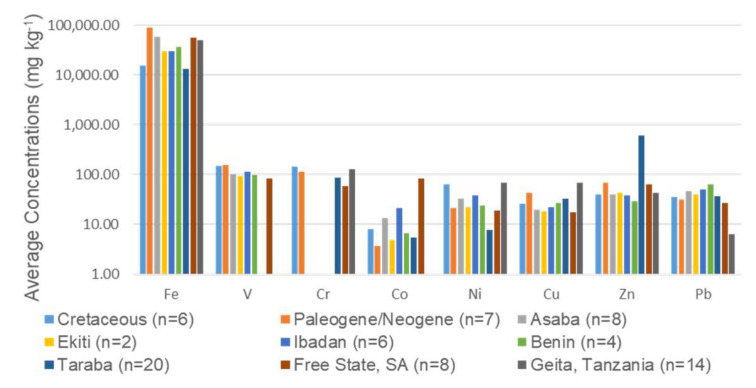
Plot of average trace element concentrations in Cretaceous and Paleogene/Neogene geophagic kaolinitic clays from Eastern Dahomey and Niger Delta Basins, Nigeria (this study) and from other studies.

Based on the trace element concentrations, the order of trace elements in the Cretaceous geophagic kaolinitic clays was Fe > V > Cr > Ni > Zn > Pb > Cu > Co, whereas in the Paleogene/Neogene geophagic kaolinitic clays was Fe > V > Cr > Zn > Cu > Pb > Ni > Co (Figure 4). Iron is the most abundant element in the geophagic kaolinitic clays which could be attributed to its abundance in the upper continental crust and the presence of hematite, goethite, and anatase minerals in them [30,51]. In addition, some of the Fe were present in the structure of the kaolinites [52,53]. 

Comparison between the trace elements in the Cretaceous and Paleogene/Neogene geophagic kaolinitic clays showed that the differences in the concentrations were not statistically significant (*p* > 0.05) except for Fe (Table 3 and Figure 4). The mean concentrations of V, Cr, Co (except geophagic clays from Asaba, Ibadan, and Free State), Ni (except geophagic clays from Geita), and Cu were higher and Fe (except geophagic clays from Taraba), Zn (except geophagic clays from Ibadan and Benin), and Pb (except geophagic clays from Free State and Geita) were lower in the Cretaceous geophagic kaolinitic clays than those reported for geophagic clays from Asaba, Ekiti, Ibadan, Benin (Nigeria: [4]), Free State (South Africa: [49]), and Geita (Tanzania: [50]) (Table 2 and Figure 4). Paleogene/Neogene geophagic kaolinitic clays have a higher average Fe, V, Cr (except geophagic materials from Geita), Cu (except geophagic clays from Geita), Zn (except geophagic clays from Taraba), and a lower average Co, Ni (except geophagic clays from Taraba and Free State), and Pb (except geophagic clays from Free State and Geita) relative to those reported from Asaba, Ekiti, Ibadan, Benin (Nigeria: [4]), Free State (South Africa: [49]), and Geita (Tanzania: [50]) (Table 2 and Figure 4).

The elements present in the geophagic kaolinitic clays consumed determine the nutritional value. The recommended dietary intake (RDI) contributions for Cr, Cu, Zn, and Fe are presented in Table 4 for the various age groups. The average contributions for Cr, Cu, Zn, and Fe were 0.34 and 0.43, 0.13 and 0.08, 0.21 and 0.12, and 272.06 and 46.68 mg/30 g, respectively for Cretaceous and Paleogene/Neogene geophagic kaolinitic clays (Table 4). The contributions from Cr and Fe were more than the RDI, whereas Cu and Zn were less than the RDI for all the various groups [54] (Table 4). This suggests that the consumption of the geophagic kaolinitic clays will not contribute to the nutrient demands for Cu and Zu of the geophagic individual.

### 3.1. Pollution and Risk Assessment

**Figure 5:**
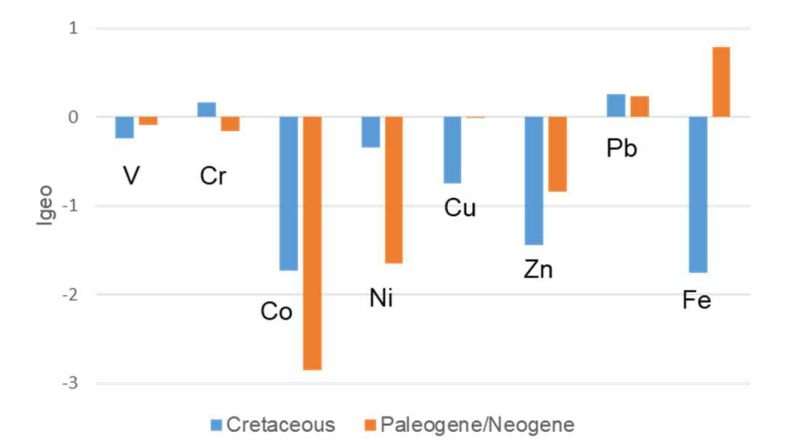
Plot of the average geoaccumulation index (I_geo_) for trace elements in the Cretaceous and Paleogene/Neogene geophagic kaolinitic clays within Eastern Dahomey and Niger Delta Basins, Nigeria.

The average I_geo_ values (Table 2) for the individual trace elements in the Cretaceous and Paleogene/Neogene geophagic kaolinitic clays were generally below zero for V, Cr (except for Cretaceous geophagic kaolinitic clays), Co, Ni, Cu, Zn, and Fe (except for Paleogene/Neogene geophagic kaolinitic clays) indicating no contamination. However, the average I_geo_ values above zero for Fe (except for Cretaceous geophagic kaolinitic clays), Pb, and Cr (except Paleogene/Neogene geophagic kaolinitic clays) for Cretaceous and Paleogene/Neogene geophagic kaolinitic clays, respectively, indicates moderate contamination levels (Figure 5). The order of the average I_geo_ values follow Pb > Cr > V > Ni > Cu > Zn > Co > Fe and Fe > Pb > Cu > V > Cr > Ni > Zn > Co for Cretaceous and Paleogene/Neogene geophagic kaolinitic clays, respectively. Apart from the earlier reasons stated for occurrence of Fe in the geophagic kaolinitic clays, the Paleogene/Neogene geophagic kaolinitic clays have higher Fe concentrations relative to the Cretaceous geophagic kaolinitic clays because of the leaching of Fe from the overlying ferricitic layer above the profiles (Figure 1) [53,55]. Lead can occur naturally in kaolinitic clays by inheritance from parent material through weathering, whereas anthropogenic sources for Pb are from mining and industrial activities. Atmospheric Pb deposition from the burning of leaded gasoline is very likely to account for the Pb in the kaolinitic clays [56].

#### 3.1.1. Non-Carcinogenic Risk

The hazard index (HI) values for the trace elements in the Cretaceous and Paleogene/Neogene geophagic kaolinitic clays through ingestion, inhaling, and dermal contact with children and adults are presented in Table 5. The HQ through the three pathways did not exceed 1 for children (except HQ ingestion in children) and adults.

**Figure 6:**
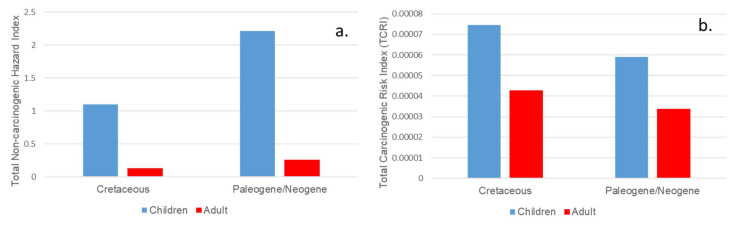
Plot of average total non-carcinogenic risk **(a)** and carcinogenic risk **(b)** for children and adults for trace elements in Cretaceous and Paleogene/Neogene geophagic kaolinitic clays within Eastern Dahomey and Niger Delta Basins, Nigeria.

This result showed that health risk associated with the consumption of the geophagic kaolinitic clays are higher through ingestion for children. The HQ_ing_ of Cr and Fe accounted for 56.44% and 26.73%, and 20.99% and 72.30% of total HQ_ing_ for children exposed to the Cretaceous and Paleogene/Neogene geophagic kaolinitic clays, respectively. From the total non-carcinogenic HI, children are in higher risk relative to adults with HI > 1 as stipulated by USEPA [57] (Table 5 and Figure 6a) when they consume the geophagic kaolinitic clays. This observation is like previous reports from other studies in Australia [58], Malaysia [59], South China [42], and Iran [26]. The consumption of the Paleogene/Neogene geophagic kaolinitic clays is riskier for the children than Cretaceous geophagic kaolinitic clays (Figure 6a). This can be attributed to the variations in the physiological parameters between the children and adult population [42,47].

The HI values of the trace elements were in the following increasing order: Cr > Fe > Pb > Ni > Cu > Co > Zn and Fe > Cr > Pb > Cu > Ni > Zn > Co for both children and adults consuming the Cretaceous and Paleogene/Neogene geophagic kaolinitic clays, respectively. It is obvious that Cr and Fe are the most hazardous elements that pose non-carcinogenic risk. This is consistent with the previous observation in this study, that Cr and Fe were present in the geophagic kaolinitic clays above the RDI. The utilisable fraction of trace elements by children and adults consuming the geophagic material will be dependent on their bioavailability and absorption in the gastrointestinal tract [5]. Excessive dosages of Fe can cause vomiting and diarrhea at low overdosage and could eventually lead to systemic toxicity at high overdosage [54]. The toxicity level of hexavalent chromium (IV) (Cr^6+^) is higher relative to trivalent chromium (Cr^3+^). In addition, Cr^6+^ is believed to become reduced into Cr^3+^ in the human body [26,60]. Evidence for the carcinogenicity of Cr^3+^ in humans is lacking, but it could cause skin rashes [54].

#### 3.1.2. Carcinogenic Risk

The carcinogenic risk associated with the Cr, Ni, and Pb in the Cretaceous and Paleogene/Neogene geophagic kaolinitic clays are presented in Table 5. CRI values from ingestion (CRI_ing_) and inhalation (CRI_inh_) for children and adults in Cretaceous geophagic kaolinitic clays were 7.40 × 10^−5^ and 4.49 × 10^−7^ and 4.23 × 10^−5^ and 5.14 × 10^−7^, respectively. In the Paleogene/Neogene geophagic kaolinitic clays, the CRI_ing_ and CRI_inh_ were 5.85 × 10^−6^ and 3.54 × 10^−7^ for children, and 3.35 × 10^−5^ and 4.04 × 10^−7^ for adults, respectively. The CRI_ing_ contributed about 99.40% and 98.80% to the total CRI (TCRI) values relative to CRI_inh_ contributing about 0.6% and 1.19% for children and adults, respectively. The TCRI for children and adults were 7.45 × 10^−5^ and 4.28 × 10^−5^ for Cretaceous geophagic kaolinitic clays and 5.89 × 10^−5^ and 3.39 × 10^−5^ for Paleogene/Neogene geophagic kaolinitic clays, respectively. The children have higher TCRI values relative to the adults (Figure 6b) which further suggests higher susceptibility of children to potential risk associated to the trace elements present in the geophagic kaolinitic clays. Risk level ranges from previous study are as follows: very low (<10^−6^), low (10^−6^–10^−5^), medium (10^−5^–10^−4^), high (10^−4^–10^−3^), and very high (>10^−3^) [47,61]. This suggests low to medium carcinogenic risk to the children and adult health consuming the geophagic kaolinitic clays. However, the TCRI values obtained are within the tolerable CRI, i.e., 10^−6^ < CRI < 10^−4^ [43,48].

## 4. Conclusions

The potential health risks due to selected trace elements in Cretaceous and Paleogene/Neogene geophagic kaolinitic clays within Eastern Dahomey and Niger Delta Basins, Nigeria have been assessed. The average trace elements concentrations in the Cretaceous and Paleogene/Neogene geophagic kaolinitic clays were 15,560.22 and 90,685.70, 149.82 and 155.08, 143.54 and 113.45, 7.92 and 3.7, 62.42 and 21.68, 26.17 and 43.80, 39.91 and 69.85, 35.16 and 30.96 ppm, for Fe, V, Cr, Co, Ni, Cu, Zn, and Pb, respectively. No significant statistical differences were observed between their concentrations except for Fe. This was attributed to the leaching of Fe down to the underlying Paleogene/Neogene geophagic kaolinitic clays from the overlying ferricretic layer. The average nutritional values for Cu and Zn were lower than RDI, whereas Cr and Fe were higher relative to the RDI in the Cretaceous and Paleogene/Neogene geophagic kaolinitic clays. The contamination levels for the trace elements were generally low except for Cr, Pb, and Fe with moderate contaminations. The Paleogene/Neogene geophagic kaolinitic clays (average HI = 2.21) pose higher non-carcinogenic risk to children relative to the Cretaceous geophagic kaolinitic clays (average HI = 1.10), particularly due to Cr and Fe (through ingestion). The carcinogenic risk to children and adults consuming the geophagic kaolinitic clays is low and generally within the tolerable limit (10^−6^ < CRI < 10^−4^). The study therefore recommends the processing of the geophagic kaolinitic clays by reducing the trace elements to acceptable levels prior to ingestion to minimise the potential human health risk particularly to children. 

## Figures and Tables

**Table:** 

(a)			
Parameters	Definition	Unit	Values Used
C	Element content in soil	mg kg^−1^	
CF ^a^	Conversion factor	kg mg^−1^	10^−6^
EF ^a^	Exposure frequency	d yr^−1^	350
ED ^a^	Exposure duration	yr	6 for children, 30 for adults
BW ^a^	Body weight	kg	16 for children, 70 for adults
AT_nc_^b^	Average time for non-carcinogenic effects	d	2190 for children, 10,950 for adults
AT_ca_^b^	Average time for carcinogenic effects	d	25,550
Ingestion			
Ing*R* ^b^	Ingestion rate	mg d^−1^	200 for children, 100 for adults
Inhalation			
Inh*R* ^b^	Respiratory rate	m^3^ d^−1^	20
PEF ^b^	Particulate emission factor	m^3^ kg^−1^	1.39 × 10^9^
Dermal contact			
SA ^b^	Skin Surface Area	cm^2^	2800 for children, 5700 for adults
AF ^b^	Adherence factor	mg cm^−2^ d^−1^	0.2 for children, 0.07 for adults
ABS ^c^	Absorption factor	Unitless	0.001 for all metals

^a^ [24,41]; ^b^ [43]; ^c^ [45,46].

**Table:** 

(b)							
	Fe ^d^	Cr ^e^	Co ^d^	Pb ^e^	Cu ^e^	Zn ^e^	Ni ^e^
*RfD*_ing_ (mg kg d^−1^)	0.7	3.00 × 10^−3^	0.02	3.50 × 10^−3^	0.04	0.3	0.02
*RfD*_derm_ (mg kg d^−1^)	-	6.00 × 10^−5^	-	5.25 × 10^−4^	0.12	0.06	5.40 × 10^−3^
*RfD*_inh_ (mg kg d^−1^)	-	2.86 × 10^−5^	-	3.52 × 10^−3^	0.04	0.3	9.00 × 10^−5^
*SF* for ingestion (kg d mg^−1^)	-	0.5	-	8.50 × 10^−3^	-	-	-
*SF* for inhalation (kg d mg^−1^)	-	42	-	-	-	-	0.84

*RfD*—reference dose; SF—slope factor; ^d^ [47]; ^e^ [48]; (-) not available.

**Table 2:** Trace element concentrations (mg kg^−1^) and average I_geo_ of Cretaceous and Paleogene/Neogene geophagic clays from Eastern Dahomey and Niger Delta Basins, Nigeria, and averages for other studies from Nigeria, South Africa, and Tanzania.

	Fe	V	Cr	Co	Ni	Cu	Zn	Pb
**Cretaceous**								
LP1 0 m	10,580.00	87.16	91.66	5.69	31.14	16.95	36.59	40.20
LP1 2 m	9722.16	172.13	136.80	12.18	52.32	49.86	42.64	18.97
LP1 4 m	38,602.70	114.00	138.81	8.61	50.13	15.84	51.76	23.96
LP2 0 m	17,442.70	305.35	218.84	7.85	164.23	44.95	46.49	28.23
LP2 2 m	8578.38	114.86	144.15	5.91	40.39	11.09	30.74	18.93
LP2 4 m	8435.40	105.44	131.02	7.31	36.36	18.36	31.27	80.69
Min	8435.40	87.16	91.66	5.69	31.14	11.09	30.74	18.93
Max	38,602.70	305.35	218.84	12.18	164.23	49.86	51.76	80.69
Average	15,560.22	149.82	143.54	7.92	62.42	26.17	39.91	35.16
Average I_geo_	−1.75	−0.24	0.16	−1.74	−0.34	−0.75	−1.44	0.25
**Paleogene/Neogene**								
UL1 0 m	66,196.48	99.94	100.86	2.47	16.14	22.96	37.60	25.66
UL1 1 m	109,374.31	177.19	124.39	3.89	30.33	37.36	48.19	41.14
UL1 2 m	74,774.85	136.42	93.17	2.73	16.46	27.64	63.23	25.13
UL2 0 m	71,200.53	161.91	127.05	2.79	20.97	17.13	32.09	36.36
UL2 1 m	95,648.90	214.78	163.06	3.12	23.52	102.97	38.73	42.69
UL2 2 m	98,937.28	113.50	88.97	4.13	16.60	46.49	132.30	19.63
UL2 3 m	118,667.55	181.83	96.66	7.25	27.73	52.08	136.83	26.10
Min	66,196.48	99.94	88.97	2.47	16.14	17.13	32.09	19.63
Max	118,667.55	214.78	163.06	7.25	30.33	102.97	136.83	42.69
Average	90,685.70	155.08	113.45	3.77	21.68	43.80	69.85	30.96
Average I_geo_	0.79	−0.09	−0.16	−2.85	−1.65	−0.01	−0.84	0.23
**Other studies**								
Asaba (n = 8) ^1^	58,618.91	102.12	-	13.56	33.16	19.88	40.40	46.30
Ekiti (n = 2) ^1^	30,167.29	95.00	-	4.90	21.85	18.56	42.30	39.53
Ibadan (n = 6) ^1^	29,881.35	115.17	-	21.57	38.13	22.04	38.32	49.69
Benin (n = 4) ^1^	36,172.16	97.50	-	6.55	24.08	26.89	29.10	64.62
Taraba (n = 20) ^2^	13,153.65	-	86.57	5.54	7.76	32.31	600.65	36.78
Free State, SA (n = 8) ^3^	56,617.29	83.62	58.62	83.30	18.86	17.24	64.00	27.38
Geita, Tanzania (n = 14) ^4^	49,771.00	-	129.00	-	69.10	67.70	43.70	6.50

(-) Not detected; ^1^ [4]; ^2^ [8]; ^3^ [49]; ^4^ [50].

**Table 3:** T-test for significance of difference in trace element concentrations (mg kg^−1^) between Cretaceous (n = 6) and Paleogene/Neogene (n = 7) geophagic Kaolinitic Clays from Eastern Dahomey and Niger Delta Basins, Nigeria (at 5% significant level).

Element		Mean	SD	t-Value	*p*-Value
Fe	Cretaceous	15,560.22	11,771.95	−8.32	0.01
	Paleogene/Neogene	90,685.70	20,238.24		
V	Cretaceous	149.82	81.35	−0.14	0.89
	Paleogene/Neogene	155.08	40.69		
Cr	Cretaceous	143.54	41.44	1.53	0.16
	Paleogene/Neogene	113.45	26.51		
Co	Cretaceous	7.92	2.37	3.61	0.06
	Paleogene/Neogene	3.77	1.65		
Ni	Cretaceous	62.42	50.52	1.98	0.11
	Paleogene/Neogene	21.68	5.76		
Cu	Cretaceous	26.17	16.69	−1.37	0.20
	Paleogene/Neogene	43.80	28.94		
Zn	Cretaceous	39.91	8.49	−1.71	0.13
	Paleogene/Neogene	69.85	45.34		
Pb	Cretaceous	35.16	23.65	0.41	0.70
	Paleogene/Neogene	30.96	8.99		

SD—standard deviation.

**Table 4:** Recommended dietary intake (RDI; [54]) and the average nutritional value of Cr, Cu, Zn, and Fe in the Cretaceous and Paleogene/Neogene geophagic kaolinitic clays, Nigeria.

Group	Range in Years	RDI (mg/d)			
		Cr	Cu	Zn	Fe
Childhood	0–8	0.0002–0.015	0.2–0.44	2–5	0.27–11
Adolescent	9–18	0.025–0.035	0.7–0.89	8–11	8–11
Adult	>19	0.03–0.035	0.9	11	8
Average Nutritional Value (mg/30 g) *					
Cretaceous (n = 6)		0.43	0.08	0.12	46.68
Paleogene/Neogene (n = 7)		0.34	0.13	0.21	272.06

* Average nutritional value = average conc. of element (wt %) × 30 g [24].

**Table 5:** Average health risks based on the average trace element concentrations in the Cretaceous and Paleogene/Neogene geophagic kaolinitic clays within Eastern Dahomey and Niger Delta Basins, Nigeria.

	Non-Carcinogenic Hazard Index															
	Cretaceous								Paleogene/Neogene							
	Children				Adult				Children				Adult			
	HQ ing	HQ derm	HQ inh	HI	HQ ing	HQ derm	HQ inh	HI	HQ ing	HQ derm	HQ inh	HI	HQ ing	HQ derm	HQ inh	HI
Cr	5.74E^−1^	8.03E^−2^	4.33E^−3^	6.58E^−1^	6.55E^−2^	1.31E^−2^	9.89E^−4^	7.96E^−2^	4.53E^−1^	6.35E^−2^	3.42E^−3^	5.20E^−1^	5.18E^−2^	1.03E^−2^	7.82E^−4^	6.29E^−2^
Ni	3.74E^−2^	3.88E^−4^	5.98E^−4^	3.84E^−2^	4.28E^−3^	6.32E^−5^	1.37E^−4^	4.48E^−3^	1.30E^−2^	1.35E^−4^	2.08E^−4^	1.33E^−2^	1.48E^−3^	2.19E^−5^	4.75E^−5^	1.55E^−3^
Zn	1.59E^−3^	2.23E^−5^	1.15E-07	1.62E^−3^	1.82E^−4^	3.64E-06	2.62E^−8^	1.86E^−4^	2.79E^−3^	3.91E^−5^	2.01E^−7^	2.83E^−3^	3.19E^−4^	6.36E^−6^	4.59E^−8^	3.25E^−4^
Pb	1.20E^−1^	2.25E^−3^	8.61E-06	1.23E^−1^	1.38E^−2^	3.66E^−4^	1.97E^−6^	1.41E^−2^	1.06E^−1^	1.98E^−3^	7.58E^−6^	1.08E^−1^	1.21E^−2^	3.22E^−4^	1.73E^−6^	1.24E^−2^
Co	4.75E^−3^			4.75E^−3^	5.42E^−4^			5.42E^−4^	2.26E^−3^			2.26E^−3^	2.58E^−4^			2.58E^−4^
Cu	8.46E^−3^	7.32E^−6^	5.64E^−7^	8.46E^−3^	9.66E^−4^	1.19E^−6^	1.29E^−7^	9.68E^−4^	1.42E^−2^	1.23E^−5^	9.44E^−7^	1.42E^−2^	1.62E^−3^	1.995E^−6^	2.16E^−7^	1.62E^−3^
Fe	2.66E^−1^			2.66E^−1^	3.05E^−2^			3.05E^−2^	1.55E^+^			1.55E^+^	1.77E^−1^			1.77E^−1^
Total	1.01E^+^	8.30E^−2^	4.93E^−3^	1.10E^+^	1.16E^−1^	1.35E^−2^	1.13E^−3^	1.30E^−1^	2.14E^+^	6.56E^−2^	3.64E^−3^	2.21E^+^	2.45E^−1^	1.07E^−2^	8.31E^−4^	2.57E^−1^
	Carcinogenic Risk Index															
	Cretaceous						Paleogene/Neogene									
	Children			Adult			Children			Adult						
	CRI ing	CRI inh		CRI ing	CRI inh		CRI ing	CRI inh		CRI ing	CRI inh					
Cr	7.37E^−5^	4.46E^−7^	7.42E^−5^	4.21E^−5^	5.09E^−7^	4.26E^−5^	5.83E^−5^	3.5E^−7^	5.86E^−5^	3.33E^−5^	4.03E^−7^	3.37E^−5^				
Ni		3.88E^−9^	3.88E^−9^		4.43E^−9^	4.43E^−9^		1.3E^−9^	1.35E^−9^		1.54E^−9^	1.54E^−9^				
Pb	3.07E^−7^		3.07E^−7^	1.75E^−7^		1.75E^−7^	2.7E^−7^		2.7E^−7^	1.54E^−7^		1.54E^−7^				
Total	7.4E^−5^	4.49E^−7^	7.45E^−5^	4.23E^−5^	5.14E^−7^	4.28E^−5^	5.85E^−5^	3.5E^−7^	5.89E^−5^	3.35E^−5^	4.04E^−7^	3.39E^−5^				
